# Aero-Medical Evacuation during SARS-CoV-2 Pandemic: Extraordinary Measure or Emerging Treatment Option?

**DOI:** 10.3390/jcm12010133

**Published:** 2022-12-24

**Authors:** Domenico Benvenuto, Tommaso Ascoli Bartoli, Ambrogio Curtolo, Claudia Palazzolo, Serena Vita, Andrea Mariano, Laura Scorzolini, Giuseppe Ippolito, Luisa Marchioni, Federico Cerini, Gianpiero D’Offizi, Francesco Vaia, Emanuele Nicastri

**Affiliations:** 1 Istituto Nazionale per le Malattie Infettive “L. Spallanzani”, IRCCS, Via Portuense 292, 00149 Rome, Italy; 2 Italian Air Force, AeroMedical Department, Pratica di Mare Air Force Base, Via Pratica di Mare 45, 00040 Rome, Italy

**Keywords:** medical evacuation, COVID-19, infectious diseases

## Abstract

Aero-medical evacuation has been considered as a feasible and safe treatment option during COVID pandemic, particularly when the needs of affected patients exceed what local clinics and hospitals are supposed to provide. In this article, we analyzed the clinical course of 17 patients medically evacuated to the “L. Spallanzani” Institute in Rome, Italy from foreign countries, mainly Africa and Eastern Europe, who had COVID-19 pneumonia with, or without, coinfections such as malaria, HIV, tuberculosis and microbiologically confirmed sepsis syndrome. The aero-medical evacuation of patients with infectious diseases has become one of the greatest medical achievements we have reached during this pandemic; in fact, only two patients with life threatening coinfections have died. Although logistically difficult and cost consuming, medical evacuation should be considered as a treatment option more than a single extraordinary measure, especially among complex cases that require specific technical and human resources.

## 1. Introduction

Aero-medical evacuation to the nearest well-equipped medical facility has proven to be extremely useful, especially when the needs of injured or ill patients exceed what local clinics and hospitals can provide [1]. The severe acute respiratory syndrome coronavirus 2 (SARS-CoV-2) pandemic has posed a serious threat for global public health, requiring great efforts to design appropriate treatment and management protocols. At the very beginning of the first pandemic wave very little was known about how to effectively treat this novel coronavirus. The fast-growing number of cases worldwide required quick decisions and one of the best ways to overcome the lack of knowledge, structures, drugs and equipment was aero-medical evacuation. The strategic position of Italy in the center of the Mediterranean basin has played a fundamental role historically; moreover, now as then, it is equidistant from Africa and Eastern Europe, making the connection between Italy and these macro-regions rather simple. In this article, we describe 17 complex cases of coronavirus disease (COVID-19) that required medical evacuation because of the lack of knowledge and/or technical and human resources in the local hospitals. Time is ripe for the development of operational standards and consensus guidelines involving Aeromedical Evacuation and High-Level Containment Transport (AE-HLCT), as suggested by Gibbs et al. 2019 [2].

## 2. Materials and Methods

This single-center retrospective longitudinal study has been performed including 17 patients with COVID-19, medically evacuated from foreign non-European countries via private flight companies or by the means of the Aeromedical Department of the Italian Air Force. All the patients were Italian citizens medically evacuated to the Spallanzani Institute from August 2020 to August 2021 and have been consecutively enrolled into the study database. The biocontainment team was composed of three doctors and five nurses with the support of logistics personnel operating at Pratica di Mare Military Airport. Within 8 hours of the "Notice to Move", the team were on board and ready for the mission. In particularly severe cases, the team also operated with a readiness of less than 4 hours. The air transport was performed by the means of isolation chambers (ATIsol) supported by a rack with electro-medical equipment capable of assisting the patient in intensive monitoring.

The procedures for the activation, readiness and conduct of the mission in biocontainment were performed referring to the Air Force Directive. During the operations in biocontainment, anesthesiologists performed resuscitation and/or resuscitation maneuvers using side sleeves, without direct contact with the patient. On arrival, the patients were taken over by the biocontainment team inside the isolator, monitored and assisted during both the boarding and flight phases. The staff responsible for the reception of the patients and proceeding to embarkation in the isolator were subjected to a strict decontamination phase carried out by three operators of the biocontainment team; a similar procedure was carried out once the patient was handed over to the health team of the accepting facility. At the end of the mission, the isolator and its internal parts underwent a strict decontamination process and technical monitoring. A Boeing KC767 in a medical wagon configuration was used for the medical evacuation; in this asset the vehicle could contain up to a maximum of 10 isolators for stable patients or 5 isolators in an intensive configuration for critical patients. We retrospectively collected anagraphic, clinical and pathological data from clinical records. Descriptive analysis was performed using GraphPad Prism statistical Software v.8.4.3 for Windows, GraphPad Software, La Jolla, CA, USA, www.graphpad.com. Informed consent was obtained from patients or their family members. All data were collected anonymously.

## 3. Results

**Figure 1:**
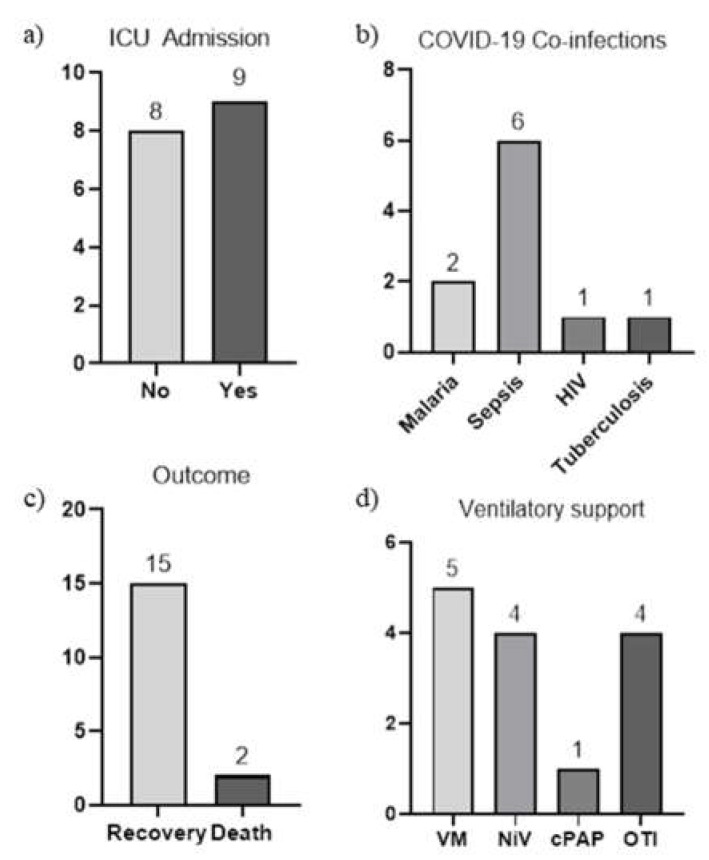
(**a**) Number of patients admitted to ICU; (**b**) Types of coinfections; (**c**) Clinical outcome; (**d**) Types of ventilatory support administered.

A total of 17 patients (5 females and 12 males) were medically evacuated to the Italian National Institute for Infectious Diseases in Rome, Italy, from foreign countries: 4 cases from Albania, 3 from Nigeria, 2 from Libya and 1 from the Congo, Kenya, Angola, Kazakhstan, Zimbabwe, Egypt and Romania, respectively (Table 1). Among the 17 patients, 12 patients had or developed coinfections or infection-related syndromes. One had a coinfection with HIV, one had a suspected coinfection with tuberculosis, two had a coinfection with malaria and six of them already had, or developed during the hospital stay, a microbiologically confirmed sepsis syndrome (three of them related to central venous catheter) (Figure 1).

Eight of them were immediately admitted to the intensive care unit (ICU) for acute respiratory distress syndrome (ARDS) while one patient was admitted to ICU ten days later, after the acute worsening of pulmonary gas exchange (Figure 1).

All patients were treated with systemic corticosteroids (oral or intravenous dexamethasone, 6 mg per day, or, as an alternative, methylprednisolone (0.5–1 mg/kg per day), anticoagulant prophylaxis (subcutaneous enoxaparin 4000 IU/mL per day) or therapy (subcutaneous enoxaparin 100 IU/Ml twice per day), antivirals (remdesevir 200 mg on day 1 followed by 100 mg on days 2–4 if within day 10 from symptoms’ onset), antibiotic therapy in the case of microbiological or imaging evidence of bacterial coinfection and with oxygen therapy support. Five patients received oxygen therapy by Venturi mask (VM) only, while two patients underwent helmet continuous positive airway pressure (C-PAP), three patients underwent non-invasive ventilation (NiV) and seven patients underwent oro-tracheal intubation (OTI) (Figure 1). The mean OTI duration was 30 days (95%CI 1–61 days), while the mean duration of the ICU length of stay was 25 days (95%CI 11–39 days). The mean age at the time of the admission was 57 years (95%CI 50–65 years) and the mean length of hospitalization was 24 days (95%CI 13–36 days). Two of the seventeen patients unfortunately died, one from severe sepsis due to multi-drug-resistant microorganisms, and the second one from rapidly progressive ARDS (Figure 1).

Other specific therapeutic regimens were required in the following patients:

- A 57 year-old male patient treated, very early in the first phase of the COVID pandemic, with three infusions of 250 mL hyperimmune convalescent plasma with >1: 320 neutralizing antibody titer against SARS-CoV-2.
- A 64 year-old female patient treated with three infusions of 250 ml hyperimmune convalescent plasma with >1:320 neutralizing antibody titer against SARS-CoV-2 and with oral acyclovir (800 mg thrice per day) for Herpes Virus—2 reactivation.
- A 50 year-old male, treated with iv tocilizumab (8 mg followed by a second infusion, 12 h apart).
- A 57-year-old patient, treated with highly active antiretroviral therapy with an oral fixed combination of emtricitabine, rilpivirine and tenofovir alafenamide.
- A 67 year-old patient, treated for severe ARDS and for polymicrobial sepsis sustained by pseudomonas, aspergillus, pneumocystis pneumonia (PCP) and cytomegalovirus (CMV), candida tropicalis and a vancomycin-resistant enterococcus (VRE). He was treated with i.v. linezolid (600 mg every 12 h), merrem (2 g every 8 h), colistin (9,000,000 I.U./daily), daptomycin (500 mg/daily), ganciclovir, anidulafungin (an initial dose 200 mg followed by 100 mg/daily), voriconazole (an initial dose of 400 mg every 12 h for the first 24 h followed by 200 mg every 12 h), Tigecycline (an initial dose of 100 mg, followed by 50 mg every 12 h), Ampicillin (2 g every 6 h) and trimethoprim (100 mg every 12 h) but unfortunately died.
- Two young males (23 and 30 years old, respectively) traveling in West Africa with no malaria prophylaxis, initially admitted to a local hospital for uncomplicated Plasmodium falciparum malaria and COVID-19 coinfection. They were treated with oral artemisinin-based combination therapy (ACT) with 20/120 mg artemether/lumefantrine, four tablets twice-daily for three days; while in Italy they received oral dexamethasone (6 mg per day) and oxygen therapy for COVID-19 pneumonia only. Two weeks after discharge, the older patient was re-admitted for uncomplicated P. falciparum malaria relapse. He was re-treated with a three-day course of ACT including 320/40 mg piperaquine/artenimol four tablets per day for three days with full recovery at the follow-up visit.
- A 59 year-old patient, admitted for malaria and COVID-19 coinfection. He was locally treated with unspecified ACT antimalarial oral therapy for 3 days, methylprednisolone (1 mg/Kg for 5 days), enoxaparin (6000 IU every 12 hours for 22 days), ceftriaxone (2 g daily for 5 days) and oxygen therapy. For the worsening of the respiratory function 12 hours after his admission, he began therapy with C-PAP for 2 weeks. During the length of stay, there was a progressive improvement until discharge with full recovery.

No cases of SARS-CoV-2 infection among the biocontainment team personnel were registered during or after the medical evacuation procedures.

## 4. Discussion

Although the SARS-CoV-2 pandemic has caused many problems, it has also accelerated medical research and the development of new effective treatment protocols. We fully understood the relevant role played by medical evacuation for severe and complicated COVID-19 cases, especially during the first wave, when limited scientific medical evidence on the clinical management of COVID-19 inpatients was available. Moreover, most cases often had a complex clinical presentation with coinfections and/or several comorbidities requiring a multidisciplinary approach. A recent review has highlighted the need for a good understanding of flight physiology and of the potential risks of a transcontinental flight [3]. Certainly, before considering medical evacuation as a therapeutic approach, the risks related to the duration of the flight should be taken into account Even though we could not predict the clinical outcome that the patients could have had without the medical evacuation, the opportunity to safely evacuate patients with infectious diseases in a high containment setting is a significant achievement and it will become even more relevant to meet the challenges presented by any future high-consequence infectious diseases [4]. Moreover, the ATIsol has proven to be a reliable tool capable of keeping patients isolated from healthcare personnel without hindering the necessary maneuvers.

The establishment of a European network for medical evacuations could significantly improve the efficacy of this methodological process, allowing us to manage difficult cases to the best of our evidence-based medical knowledge, even including experimental protocols for emerging pathogens and updating therapeutic regimens reducing the case fatality risk balance.

## 5. Conclusions

Although medical evacuation has almost 80 years of history, more efforts are still needed to completely fulfill and tailor its high potential performance. Nowadays, considering the different scenarios that the world might face, medical evacuation, although logistically difficult and expensive to organize, should be carefully evaluated as an innovative treatment option more than a single extraordinary medical measure, to increase the clinical survival outcomes of affected patients.

## Figures and Tables

**Table 1:** Gender, country of departure, hospitalization time expressed in days, age, ventilatory support, concomitant infections and clinical outcome.

Gender	Country	Hospitalization (Days)	Age (Years)	VM, cPAP, NiV, OTI ^†^	Coinfections	Clinical Outcome
F	Albania	92	64	OTI	sepsis	Recovery
M	Albania	39	50	OTI	sepsis	Recovery
F	Albania	21	78	VM		Recovery
F	Albania	38	78	OTI	sepsis	Recovery
M	Nigeria	38	67	OTI	sepsis	Death
M	Nigeria	7	63	VM	HIV ^§^	Recovery
F	DRC *	14	67	NiV	TB ^‡^	Recovery
M	Kenya	30	51	c-PAP	sepsis	Recovery
M	Libya	9	59	NiV		Recovery
M	Angola	22	59	c-PAP	malaria	Recovery
M	Kazakhstan	25	57	NiV	sepsis	Recovery
M	Nigeria	3	65	OTI		Recovery
F	Zimbabwe	16	64	OTI		Death
M	Egypt	11	55	VM		Recovery
M	Romania	24	47	OTI		Recovery
M	Libya	10	23	VM	malaria	Recovery
M	Libya	12	30	VM	malaria	Recovery

^†^ Venturi Mask (VM), Continuous Positive Airway Pressure (c-PAP), Non-invasive Ventilation (NiV), Oro-Tracheal Intubation (OTI), ^‡^ Tuberculosis (TB); ^§^ Human Immunodeficiency Virus (HIV), Democratic Republic of Congo (* DRC).

## Data Availability

Not applicable.
